# Supplementary figures and images for: Covariations between pupil diameter and supplementary eye field activity suggest a role in cognitive effort implementation
Source: PLoS Biol. 2022 May 26;20(5):e3001654. doi: 10.1371/journal.pbio.3001654 (PMC9135265; doi:10.1371/journal.pbio.3001654)

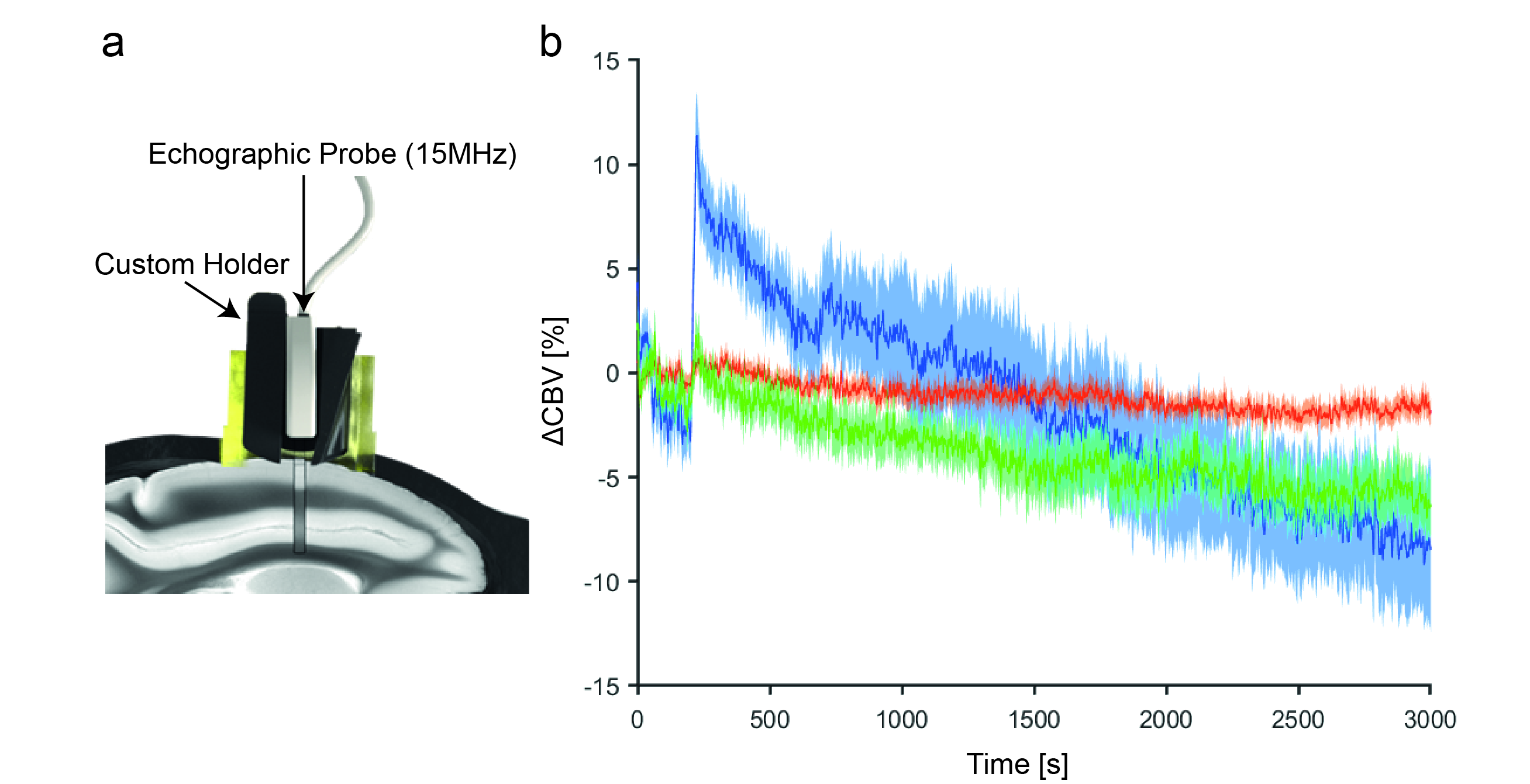

Supplement: S1 Fig — (a) Custom holder for adaptation of the ultrasonic probe (15 MHz) to the recording chamber on the animal. (b) ΔCBV for the SEF (in blue), the ACC (in red), and the control area (in green) +/− SEM across all sessions. The data underlying the graphs shown in the figure can be found in https://osf.io/2q357/. ACC, anterior cingulate cortex; CBV, cerebral blood volume; fUS, functional ultrasound; SEF, supplementary eye field. (TIF) [file pbio.3001654.s001.tif]

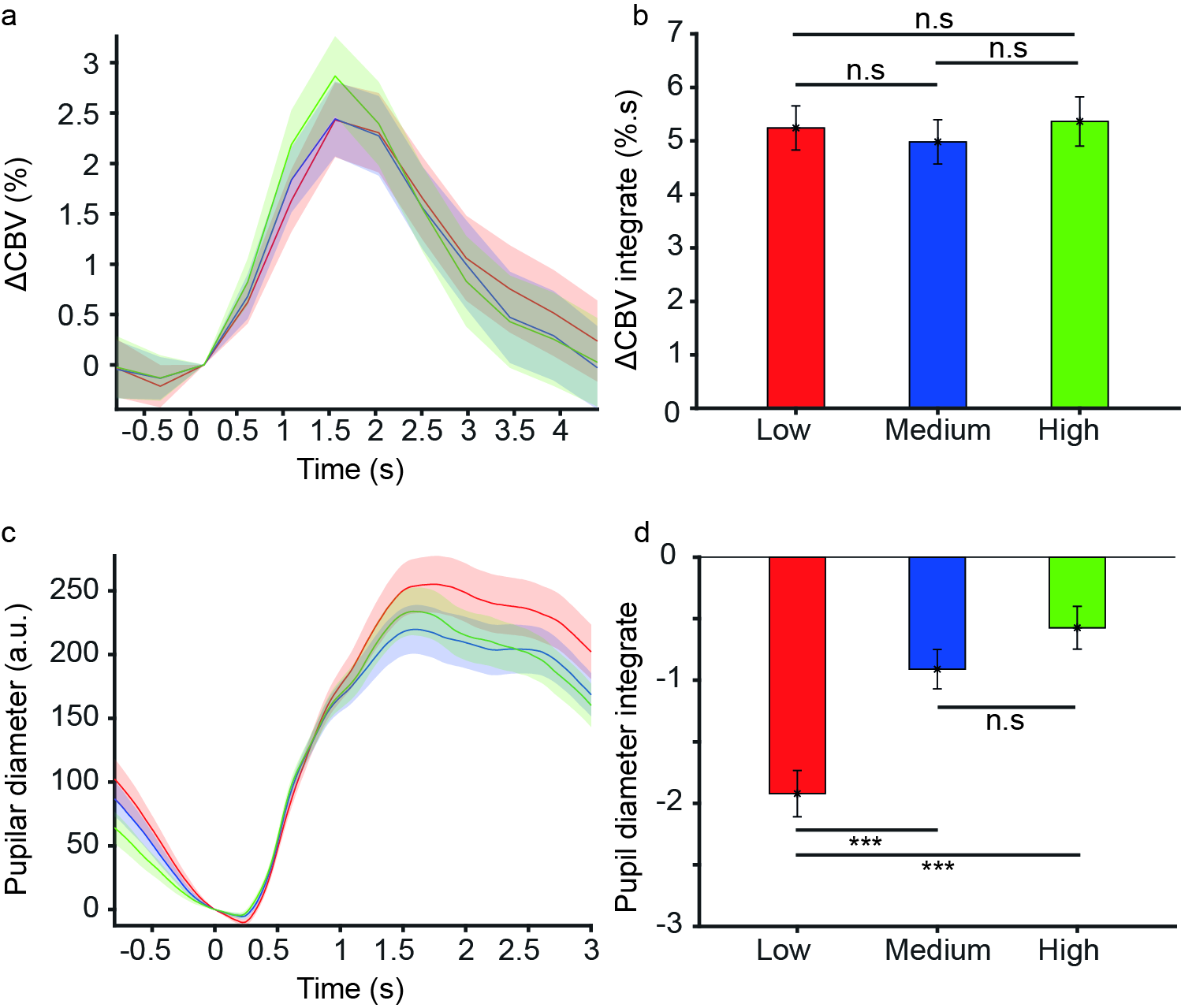

Supplement: S2 Fig — (a) Average ΔCBV response for low (red), medium (blue), and high (green) reward. (b) Integration between t = 0 s and t = 4.4 s of the previous ΔCBV curve. (c) and (d) Same for the pupil diameter. Integration is calculated between t = 0 ms and t = 320 ms. n.s.: not significant, *** p < 0.001 The data underlying the graphs shown in the figure can be found in https://osf.io/2q357/ (TIF) [file pbio.3001654.s002.tif]
