# Supplementary material for: Covariations between pupil diameter and supplementary eye field activity suggest a role in cognitive effort implementation
Source: PLoS Biol. 2022 May 26;20(5):e3001654. doi: 10.1371/journal.pbio.3001654 (PMC9135265; doi:10.1371/journal.pbio.3001654)
Supplement: Attachment — Submitted filename: Review_Claron_et_al_pupille_24012021.docx [file pbio.3001654.s003.docx]

Dear editor

We would like to thank you for considering our manuscript #pbiology-d-21-02110r1

We carefully read the four reviewers’ comments, and we sincerely thank all the reviewers for their enthusiastic examination of our original work.

Please find our comments and responses to their specific questions.

**We would like to thank reviewers #1, #2, #3 and #4 for their careful readings and their constructive commentaries. Please find our answers to comments in red as suggested text changes in blue in a new version of our manuscript.**

**Reviewer #1:** In this study, Claron et al. used functional ultrasound imaging while measuring the pupil diameter in macaque monkeys performing a saccade task to investigate the link between SEF activity and cognitive effort.

The paper is interesting, well written and overall pointing out a relationship between SEF and cognitive effort. However, the quality of the manuscript could be greatly improved by addressing several concerns.

We sincerely thank the reviewer for his/her very positive comments.

1. Further clarifications are needed regarding the data analysis. It is unclear why the values of pupillary diameter were chosen at 0.8s and 0.6s for the data analysis in Fig.2; for instance, why not using 0.8s for monkey G, since the diameter is higher than at 0.6s ?

Our selected time point of 0.6s and 0.8s of the pupil response corresponds to the first local maximum of the pupil variation as shown on figure 2.a and 2.e by the black vertical bar. However, according to reviewer's suggestion, we performed an extra analysis using both 0.6s and 0.8s as selected time points for the two animals. We presented the results in the figure R1 and we did not find substantial variations of the quantified effects	.

**Figure R1: Covariations of pupillary and vascular responses for all recording sessions for two different chosen time (0.6 s and 0.8 s, left and right column respectively) for both animals (Monkeys S and G, top and bottom rows respectively). Top right and bottom left panels are reproduction from figures 2i and 2 j. Scatter plots of the ΔCBV slope in function of pupil slope in SEF (blue), ACC (purple) et Control area (green), prediction intervals are the 95% prediction interval.**

The 1^st^ row shows graphs and correlations for Monkey S, respectively 0.6 sec and 0.8 sec (left and right graphs). We did not find any substantial variation of quantified effects: high significant effect for SEF, no effect for control area and lower significant effect for ACC (p=0.042 bot both chosen times). For Monkey G, we have noticed that the correlation in SEF was higher for 0.6 sec than for 0.8 sec with similar correlations for ACC and control areas. Given the similarity of the results using both qualitative or algorithmic approaches, we consider our time point selection using an algorithmic selection of first local maximum deflection more reproducible (0.6 s for Monkey G and 0.8 s for Monkey S) in this new version of the manuscript. However, we have clarified this methodological point and better explain our algorithmic time point selection (page 5 L.204).

Additionally, the temporal profile of the pupil size variation is different during the first trials as compared to the last trials in monkey S, making it difficult to choose a consistent time point. Therefore, these time points seem quite arbitrary and should be more justified, especially since it may have an important impact on the results

We understand the concern about the difference in the response for Monkey S between the beginning and the end for the recording session. We have now compared the first local maximum of the pupil variation for the 100 first trials and for the latter trials for 21 sessions for Monkey S. We found an average difference of 0.045 s between the first 100 trials and the latter 100 ones. We choose to compare the 1^st^-100^th^ versus 401^st^-500^th^ trials as the shortest session is a little bit over 500 trials.

Statistically speaking, the paired T-test of the medians of the 1^st^-100^th^ trials versus 401^st^ – 500^th^ trials (as data are not normal according to Shapiro-Wilk test, the median seems to us a better estimator) does not reject the null hypothesis with a p value of 0.07. So we could conclude that the temporal profile of pupil size variation were similar for the first trials and the latter ones for Monkey S.

We apologize for not having explicitly mention this point in the previous version of the manuscript (modifications page 5 l.204)

2. In this regard, the interest of using only the pupil diameter at maximum dilation for further analyses is not clear to me and should be better explained. Is this step used for all results depicted in Fig 2, and if yes, why not using all pupil diameter values (after temporal realignment and resampling with the fUS signal) ? This would enable to study the correlation between cognitive effort and SEF activity at shorter time scales occuring in a single trial (which is something discussed later but only in terms of difference between antisaccade and prosaccade: as variations of pupil size are also occurring at the trial level, it would be more convincing to take them into account).

We share the reviewer's interest in a more continuous analysis between pupil size and CBV response. The analysis produced in Figure 2 using the generalized linear model (GLM) associating the pupil response over time and the CBV response seems to answer this question. The pupillary value are extracted at the time of the fUS images (down-sampling from 1 kHz to 2.5 Hz) and then, we applied the GLM. However, an important part of the pupillary information is lost during the down-sampling of the pupil, as we are not able to detect quite rapid fluctuations at 2.5 Hz.

Regarding the within trial co-variations, the CBV measurements performed in these experiments are recorded at 2.5hz and therefore do not allow us to have enough samples (n= 13 in average points, from -1.2 to 4.0 sec around the trial) to correctly perform a GLM estimation. This point is now discussed (Page 15, L.780 to L.783) as a limitation of the present acquisitions. We hope that future recordings with higher sampling rate will be possible with a novel CBV acquisition system.

3. It would be valuable to show more results at the group level instead of examples of one session for each monkey. The supplementary figure is useful and should also include the average of all sessions for the pupil diameter variations. The variability across the Z-score maps for the different sessions should also be shown or discussed.

As shown in the figure 2.i for monkey S and 2.j for monkey G, the pupil slope is, in the vast majority of sessions, decreasing (visible on the abscissa) so is the slope for the ΔCBV (visible on the ordinate). For a clearer understanding of what is represented on 2a-d and 2e-h, a full lozenge indicates the represented sessions on the population analysis of figure 2.i and 2.j to show that our examples were representative of the full population.

We also want to highlight that we represented all the sessions (in figure 2.d for Monkey S, and 2.h for Monkey G) and we obtained the figure 2.i for Monkey S and 2.j for monkey G, indicating a slow and steady decrease of the pupillary values across a whole session, as shown on the abscissa of Fig 2.i and 2.j for all the sessions linear fit.

As suggested by the reviewer, we have plotted all Z-map for all sessions (Figure R2, at the end of the review for readability sake), both monkey S. and G. We choose not to exclude any sessions, even though we did not see activations with the generalized linear model.

4. In Figure 3, the same time scales should be used for pupil diameter and CBV in order to visualize the full temporal profiles of the responses and discuss the correlation between them at the trial level.

We agree with this comment and the figure 3 has been modified accordingly.

5. How was performed the positioning of the probe in the cranial window to select a repeatable field of view ?

**Figure R3: Custom holder for ensuring a repeatable field of view between sessions. The yellow part consists of the recording chamber, surgically fixed on the head of the animal. A custom holder, made to be inserted in the recording chamber, helps to ensure a repeatable field of view between sessions by holding the echographic probe in the same position**.

The probe was positioned in the recording chamber using an home-made adapter (represented in black in Figure R3) with a small notch allowing the probe to be positioned at the same position for each recording session. Moreover, the thickness of the imaging plane (from 700µm at the start to 400 µm at the focal plane, around 8 mm from the probe) allowed a repeatable field of view even though the position was slightly variable. This scheme is now presented in Supplementary Figure 1. Methods have been modified page 15 l.769 to l.775.

v

**Reviewer #2:** In this paper entitled 'The supplementary eye field tracks cognitive efforts', Claron et al. use fast ultrasound imaging to monitor in real-time the hemodynamic response evoked in the supplementary eye field (SEF) during the execution of a demanding cognitive task. Pupil size has been shown to track cognitive effort both at the single trial level and at the session level of such tasks. The authors thus seek to characterize the relationship between pupil size and SEF cerebral blood volume variations (CBV). They show that SEF hemodynamic activity is a robust predictor of pupil size variations at multiple time-scales including when experimentally manipulating cognitive effort and reward expectation.

The data is compelling and quite surprisingly shows that this correlation between CBV and pupil size is restricted to the SEF and does not extend into the adjacent cingulate cortex or along the adjacent cortical convexity. While this brings a very novel understanding of the physiological underpinnings of cognitive effort, it also raises a lot of crucial questions for upcoming research projects.

I thus strongly recommend the publication of this work once my comments below have been addressed (page and line numbers would have been useful to the reviewing process):

Major:

We sincerely thank the reviewer for his/her enthusiastic support to publish this study.

1.      Abstract: the authors use in their concluding statement the expression 'SEF is an underestimated but pivotal cortical area for the monitoring and implementation of cognitive effort signals': I strongly recommend the authors to reformulate as this suggests a causal role, which has actually not been demonstrated in the study. More generally speaking, I recommend the abstract to be reworked in depth as it does not make justice to the results: key concepts (e.g. cognitive effort, CBV) are not defined and the text can be clarified (e.g. 'Here, we tested […] is also recorded' appears like the same information comes up multiple times in multiple sentences).

We agree with the reviewer. Our fervour during the analysis and writing of the summary led us to some semantic excesses. We have modified the title and abstract to better reflect our contribution and conclusion in view of the existing literature.

In the new version of the abstract, we now reformulate the abstract to remove any causal effect. The main point of the abstract is more focussed on the co-variation of SEF-CBV activity and pupil activity, as assessed here: “We tested whether SEF activity is related to pupil dynamics during a mixed pro-/anti-saccade oculomotor task in two macaque monkeys” (p.2 l. 25-27).

The part “SEF is an underestimated but pivotal cortical area for the monitoring and implementation of cognitive effort signals” has been replaced by “These results bring novel understanding of the physiological underpinnings of cognitive effort involving SEF, but it also raises questions about the role of ACC” (page 2, L.36-38)

We also changed the title of the article to “ **Short and long time scales co-variations between pupil diameter and Supplementary Eye Field activity** “ as it seems to better reflect what the article is about.

2.      I recommend the authors also scrutinize the introduction in order to enhance the impact of their work. For example:

1) '..; which mediates arousal, are likely involved': references should be added;

We now add references regarding the role of the dmPFC in arousal (Page 3) :

p.3 l.99	: Ebitz, R. B. & Moore, T. Selective Modulation of the Pupil Light Reflex by Microstimulation of Prefrontal Cortex. *J. Neurosci. Off. J. Soc. Neurosci.* **37**, 5008–5018 (2017).

p.3 l.99 : Lehmann, S. J. & Corneil, B. D. Transient Pupil Dilation after Subsaccadic Microstimulation of Primate Frontal Eye Fields. *J. Neurosci. Off. J. Soc. Neurosci.* **36**, 3765–3776 (2016).

p.3 l.101 : Trommershäuser, J., Glimcher, P. W. & Gegenfurtner, K. R. Visual processing, learning and feedback in the primate eye movement system. *Trends Neurosci.* **32**, 583–590 (2009).

p.3 l.101 : Schall, J. Visuomotor Functions in the Frontal Lobe. *Annu. Rev. Vis. Sci.* **1**, 150724172340006 (2015).

2) Second paragraph 'In principle … Alternatively ….', please reformulate and back up with strong references.

We added some references to the second paragraph, as suggested:

- Parthasarathy HB, Schall JD and Graybiel AM (1992) Distributed but convergent ordering of striatal projections: The frontal eye field and the supplementary eye field in the monkey. The Journal of Neuroscience 12:4468-4488.
  - L108, page 3
- Pouget P, Stepniewska I, Crowder EA, Leslie MW, Emeric EE, Nelson MJ, Schall JD (2009) Visual and motor connectivity and the distribution of calcium-binding proteins in macaque frontal eye field: implications for saccade target selection. Frontiers in Neuroanatomy 3:2.
  - L108, page 3
- Bullier J, Schall JD and Morel A (1996) Functional streams in occipito-frontal connections in the monkey. Behavioral Brain Research 76:89-97.
  - L108, page 3
- Leichnetz, G. (1990). Preoccipital cortex receives a differential input from the frontal eye field and projects to the pretectal olivary nucleus and other visuomotor-related structures in the rhesus monkey. Visual Neuroscience, 5(2), 123-133.
  - L104, page 3
- Ebitz R.B; Moore T. Selective modulation of the pupil light reflex by microstimulation of prefrontal cortex. J. Neurosci. 2017; 37: 5008-5018
  - L99, page 3
- Ebitz R.B. Platt M.L. Neuronal activity in primate dorsal anterior cingulate cortex signals task conflict and predicts adjustments in pupil-linked arousal. Neuron. 2015; 85: 628-640
  - L104, page 3

3) 'Although the function …': I think this statement is extremely vague. The authors use anatomical evidence to suggest a role of SEF activity in the control of pupil size. This should be made explicit (control? Single controller? Why not FEF as a controller? Why not ACC?);

The text has been modified to precise our hypothesis.

“The FEF is known to be involved in the control of eye movements and attention and recent studies have shown that the amplitude of pupil responses depends on the combination of the light stimulus and subthreshold FEF electrical microstimulation^8,9^. Strongly interconnected to the FEF and anterior cingulate cortex (ACC), the SEF is a key region that integrates attentional, short-term memory, and oculomotor tasks^10,11^. The SEF also directly projects to the brainstem oculomotor nucleus. dPMC including, the ACC, SEF, and FEF networks may directly modulate the olivary pretectal nucleus, which encodes retinal illumination and directly activates the pupil-constrictor pathway^8,12–14^. In addition, the ACC, SEF, and FEF networks may act indirectly through the occipital visual cortical areas, or superior colliculus (SC), in which the visual responses are modulated by FEF and may, in addition to programming the oculomotor plan, participate in the pupil light reflex (PLR)^15–18^. Although the function of the SEF in oculomotor tasks is reasonably well defined, involvement of SEF activity in the frontal controller circuit of pupil dynamics is still unknown.” p.3 L96-110

4) 'suggesting valid qualities': please reformulate, I am not sure I get the actual meaning here;

The text has been modified (L111-114, p.3) to better explain our argument.

“Pupil dynamics have been studied during preparation and before the execution of eye movements during oculomotor protocols^19^ providing unique insights into the neuronal substrate coordinating cognitive processing, sensor-motor transformation and pupil diameter.”

5) 'Given that the SEF is known …': I am missing the logic link here: why is the fact that the SEF is crucial to anti-saccades calls to investigate pupil size in this context? This should be made explicit.

We add a sentence to better explain our argument (L147-149,p.4).

In humans, during saccade preparation, pupil size appeared to be larger in preparation for correct anti-saccades than in preparation for correct pro-saccades. Given that the SEF is known to be critically involved in the production of antisaccades^21^ […]

3.      CBV is extracted "using a random interval of 3 to 4 s after each trial": why random? Please describe trial length statistics and reward timing. It might be useful to increment the GLM with variables of non interest at this stage, such as trial outcome (i.e. reward / no reward or trial type anti- pro-saccade).

Again, we thank the reviewer for his/her remark. We would like to apologize for this misunderstanding. The CBV is acquired continuously at the rate of 2.5 Hz and extracted at each trial on  a time window of -1.2 to 4.4 sec (from target  onset). Those values were chosen as they are a multiple of 0.4 sec, which is the sampling of the fUS imaging. The random interval is referring to the inter-trial interval to avoid any anticipatory movement preparation from trial to trial. The text has been modified to better clarify this point (Page 5,L 198-199).

“Each trial is separated from one another by a random interval of 3 to 4 s, this jitter allowing avoidance of monkey anticipation between trials.”

We tried to implement the GLM with variables of non interest, such as proposed (correct versus wrong trials, pro- versus anti-saccades) but there is not enough wrong trials to make this study reliable enough. For the anti- versus pro- saccade, we also tried to do the analysis, however we did not see a difference between activated areas; we might explain the absence of difference between activated areas by the fact that ours trials are totally randomized.

4.      In section 3 of the results, better describe how many sessions were excluded per monkey. Also, rather than using an arbitrary threshold of 0.6 you might want to consider using IQR.

**Figure R4: Pupil relative diameter in function of the performance for monkey S (red triangle) and for monkey G (blue lozenge) with the old criterion of 0.6, the mean +/- 2 std and by selecting all sessions and associated p-values. (right) : same but the fUS relative response.**

Our selected performance (ratio between wrong pro-saccade and wrong anti-saccades) limit of 0.6 was manually selected. For monkey S, no sessions were excluded (0 / 20) and a total of six sessions were excluded  (6/19) for monkey G using our qualitative criteria.

We decided to not use the interquartile range because we did not want to exclude 50% of the sessions and we do not have sufficient data to, if we understood right the IQR. However, we agreed with the reviewer that the utilisation on an arbitrary threshold might be quite controversial, so we are proposing to not exclude any data from ours analysis.

5.      Section 3 is conceptually very important. What would further strengthen it is 1) to produce the equivalent of figures 2i,j,k for pro- and anti-saccades independently and 2) compare the strength of correlation between CBV and pupil for anti- and pro-saccades across sessions. The idea is to test whether correlation is strictly equivalent or is stronger in high demand trials relative to lower demand trials.

We agree with the reviewer that it would be interesting to test whether correlation is strictly equivalent or is stronger in high demand trials relative to lower demand trials (pro versus antisaccades).  Unfortunately, as our trials presentation is randomized, as a consequence we are unable to produce fig. 2 i,j,k for pro- and anti-saccades independently, as the session’s slope will remain identical. Further examination comparing long behavioural sessions of only pro or antisaccade trials will need to be recorded to fully address this question. We added a paragraph about this limitation in the results sections (p.9, L412-415)

“Between blocks of distinct reward values we did not observe significant changes in covariations between pupil and fUS responses in SEF. The duration of the periods (only 100 trials) during reward manipulation may not allow us to quantify such modulations observed at the session length (~600 trials)”

6.      Can the authors produce the plots of figure 4c,d for performance and the plots of 4e for CBV and pupil size as the information provided by each is different.

We thank the referee for their precious insight.

Figures 4.c and 4.d illustrate the transient modulations (comparing 10 trials prior a reward change to 10 trials after a reward change) in both pupil diameter and cerebral blood volume. As our trials are randomized, random generation could even make the number of antisaccades at transition less than 5 over 10 trials if pro-saccade vs. anti-saccade were evenly draw without any randomness. For those reasons, we are not able to compute the figure 4.c. and 4.d. for the performance and we would like to apologize for this.

For the 4.e. for CBV and pupil size, we are not entirely sure that informations provided by such plots will be relevant. Indeed, the position in the whole session of the different rewarded blocks are randomized, and more than one effect might be superimposing on each other, and thus, plots will be quite noisy.

However, we did the analysis as requested by the reviewer (these figures are now presented in Suppl 2), please find below the results for the CBV and the pupil.

**Figure R5 : Different vascular response for the ΔCBV in the SEF for Low (red), Medium (blue) and High (green) rewards (left) and the intensity of the response (right), modelled by the integral of the signal between t = 0 and t = 4.4 seconds. The right-most figure represent Fig 4.e. for the CBV.**

**Figure R6: Different vascular response for the ΔPupil for Low (red), Medium (blue) and High (green) rewards (left) and the intensity of the response (right), modelled by the integral of the signal between t = 0 and t = 325 ms, as described in the article. The right-most figure represent Fig 4.e. for the Pupil diameter. We do observe an effect for the low-high reward and low-medium, but nothing conclusive between medium-high. (p^low-medium^ = 1.4x10^-4^, p^low-high^ = 1.4x10^-7^, one-way ANOVA).**

For the CBV, the trial-by-trial analysis does not permit to identify a difference between the vascular responses for different reward level (Fig 3.b). However, we did identify a difference between pupillary responses for the 3 level of reward, as described in Fig 4.d.

One possible explanation for this difference might reside in the fact that neuronal substrate controlling the pupil is including the SEF but not only, and other controllers such as FEF or ACC could encode for the observed difference.

 In this context, can the authors say something about the observed saturation for both pupil size and CBV as a function of reward change. Is the coding of relative reward only qualitative?

**Figure R7: Boxplot of the Δpupil (left) and ΔCBV (right) in function of the log(ΔReward) in order to examine the variation in the pupil and CBV as a function of the Weber’s law in value perception.**

We thank the reviewer for his/her remark. Analytically, the perception of reward magnitude, time, and subjective value has been shown to change all approximately to Weber's law (REF: Namboodiri VM, Mihalas S, Hussain Shuler MG. A temporal basis for Weber's law in value perception. Front Integr Neurosci. 2014 Oct 14;8:79. doi: 10.3389/fnint.2014.00079). We might hypothesize that the saturation of both reward and pupil size for large rewards may reflect the limited delta of the subjective perception of reward observed for the largest rewards. Because our reward manipulations were limited, the sample of delta of reward as  function of reward magnitude, might not  be either correct  and certainly not conclusive. Future manipulations of volumes as well as nature of rewards might permit a  better exploration of this question. We discussed this hypothesis and the limitations of our study on page 8 in the results sections(p.8 L. 384-386)

“The observed saturation for pupil size and CBV for the largest reward changes may reflect the limited delta of the subjective perception of reward manipulated in our experiments”

Last, can the authors quantify the correlation between the delta pupil size and the delta CBV across all reward transitions and across all sessions. This would strengthen their point, all the more that the single example shown in figure 4a,b does not suggest a strict correspondence as illustrated in figure 2 in the absence of reward manipulation. The underlying question is whether the correlation between SEF CBV and pupil size is comparable across all modeled variables or whether correlation is stronger for some of them? This would help better qualify the mechanism under consideration.

The figure 4.c and 4.d has been redone showing all experimental points for the SEF and the pupil, as requested.

We thank the reviewer for his refined and precise examination of figure 4. We did not identify any significant difference in co-variations between the different reward manipulations (This information has been added in the manuscript page 9). The duration  of the periods (only 100 trials) during reward manipulation blocks may not allow us to quantify sufficiently marked variations, which is observed during the full session (more than 600 trials). Blocks with reward manipulations with ~300 trials may be sufficient but will require further explorations.  This point is discussed on p. 9 L.416-L.415

7.      Can the authors speculate on why CBV and pupil size decrease linearly when reward is constant (figure 2c,g) but not when it is not (figure 4a,b) -I apologize in advance if I missed some methodological detail here.

This is a very interesting point. Some studies have shown that, over periods of hours, days and even weeks, mental effort and cognitive fatigue progressively degrade cognitive performance (Lockley et al., 2004; Proctor et al., 1996; Virtanen et al., 2009, Cowley et al., 2020). This decrease of performance in time has been theorized in terms of neuronal and metabolic resource control (Thomson et al., 2015). “Here we report that during a sustained execution of a demanding oculomotor task over more than one hour, the neuro-vascular coupling (as assessed by fast ultrasound imaging, fUS) in the supplementary eye field (SEF) of macaques, progressively drops with time, correlating with a simultaneous drop in pupil size and performance. When changing reward ratio, intrinsic brain mechanisms might compensate for the progressive drop in cognitive performance by instantiating alternations of high and low performance. These variations in behavioural performance might be phase-locked to variations in attention- and perception-related information as shown in macaque frontal eye field (FEF) neuronal populations (Gaillard et al., 2021)”. This point is now discussed on p.11-12 L626-L665.

8.      Does covariation between SEF CBV and pupil size change between correct and incorrect trials? If this is the case, this would be quite interesting functionally speaking.

We agree with the reviewer that analyses of the comparisons between the different trials could be informative. Although somewhat at the margin of the analyses produced in this manuscript. We are currently collecting additional data (with more error trials) by modifying the task (aspect ratio) and therefore produce more error trials for the two animals. This will also allow us to quantify the effects of pass/fail sequences of trials. At this stage, we have too little evidence to describe these effects.

9.      The discussion includes an extensive discussion of human ACC. A recap of human SEF activity in effort and motivational tasks would be useful.

The remark is well taken. A paragraph on SEF has been added in the discussion. (Page 10 l.443to l.464)

« In both human and non-human primates, seminal studies have shown that SEF can indirectly influence the selection of behavior through modulation of this primary selection process in the motor structures. In humans, numerous studies suggest a role for the SEF in controlling ‘internally generated’ eye movements during the performance of complex learned behavior^25–27^ Rare studies have been capable of further examination. The role of SEF in human by examining failures in saccadic performance of patients with lesions subsuming this area^25–28^ One report from a sporadic patient with a highly focal lesion of the left SEF across a range of saccadic tasks has shown that this patient had great difficulty changing from an initial saccade plan to an alternative one using a novel change-of-plan paradigm or to reverse the rule^29,30^.Functional imaging of healthy individuals performing a change-of-plan saccadic task demonstrated enhanced SEF activity when subjects successfully changed their saccadic plans^31^. Human neuroimaging experiments show that activity levels in and around the pre- SMA increased when response speed is emphasized during speed-accuracy tradeoff experiments^32,33^. Based on these findings, it has been proposed that a significant role of the SEF lies in implementing control over conflicting internally generated saccadic plans^30,31^. In macaques, SEF also shows systematic changes in activity during learning of new stimulus-response associations^32^. »

10.     At the end of the discussion, there is a mention that CBV changes precede pupil size changes. I apologize upfront if I missed this analysis, but I couldn't find it in the manuscript. This is obviously a very useful and important information. Can you please clarify. If this is indeed the case that CBV precedes pupil size change, and depending of the latency between the two signals, the authors will want to explain if and how this impacts the reward manipulation section, as the data of both signals is sampled on the same number of trials around the time of shift in in reward size.

The authors would like to apologize for this part of the discussion; an extra analysis was included in an earlier version of the manuscript. This analysis was comparing the latency of the pupillary light response (PLR) and the hemodynamic response function (HRF) for the vascular response. However, supposing the use of the HRF seemed appropriate in the case of the vascular response, previous reviews questioned the use of PLR as an appropriate response to compare to. The text has been corrected.

11.     Overall, the manuscript should be re-read thoroughly for English phrasing and fluidity. G**enerally speaking, the impact of the work can be strengthened by initiating every result section by a statement of the question being asked (this often comes quite late in the section), and a conclusion statement wrapping out what has been demonstrated.**

We did some modification as requested:

Section 1:

‘We wished to investigate the relationship between pupil diameter and brain activity without any *a priori* choice concerning the activated area’ p.5, l.194-195

‘Finally, we can infer a similar activity in the pupil and in the SEF, as denoted by the application of the GLM’ p.6 l.224-225

Section 2:

“We are now interested in knowing if the pupil diameter and the activity in the SEF is not only having co-variation at short time scales, but also as long time scale, *id est* during a whole session.” p.6 l.232-234

“Those results seems to indicate that pupil activity and SEF activity are linked together during the time of a session.” p.7 l.290-291

Section 3:

“Given the correlation between the activity of the pupil and in the SEF, we were also interested in the difference of activity for a given cognitive load.” p.7 l.296-297

“We showed, here, that the vascular response for anti-saccades is higher than the vascular response induced by pro-saccades (p < 0.01), showing that the SEF is not only sensitive to long-term variations (minute variations) but also short-term effort-related variations (single trial variations). » p.8 l.368-371

Section 4:

“Since correlation does not imply causation, we wanted to manipulate reward to test the causality of our measures.” p.8 l.375-376

“Overall, at a transient time scale (few trials) the fUS activity in the SEF was strongly modulated in real-time by the reward, as was pupil diameter.” p.9 l.415-417

Minor:

1-      Introduction, second paragraph: replace short memory by short-term memory

This has been done.

2-      Results, title 1: I suggest something more specific for example 'Pupil size covaries with SEF CBV at short time scales'

Thanks for your suggestion, this has been done

3-      Figure 1, indicate time scale in panel a for pupil diameter and rCBV

This has been done

4-      Did the authors mean "after" rather than before Bonferroni correction in first paragraph of section 1 and elsewhere? If not, then this is a pb

Sorry for this misunderstanding. The p-value was 0.05/N with N the number of multiple comparisons. We meant the p-value before the correction was 0.05 (so approx. 4e-6 after the correction)

5-      Results: "using a random interval of 3 to 4 s after each trial ONSET"

This has been clarified after your question n°3.

6-      Results: monkey G. (Fig. 2.e) not Fig. 2.d

This has been corrected

7-      Figure 2: panels I and j : indicate monkey identity + indicate p of correlation

This has been done

8-      Results, title 2: I suggest something in the line of 'Pupil size covaries with SEF CBV at the time scale of the session'

 	Thanks for your suggestions, we choose a new title according to your suggestions.

9-      Results '… after the initial step': please specify

This has been done

10-     Results 'The decrease (no s) in pupil size correlated with the change in the activity of the SEF. THIS IS QUANTIFIED NEXT'

This has been done

11-     Results, I would place the description of the behavioral performance of the monkeys on the pro- and anti-saccades under section 3. Because that's where it is relevant. At the beginning of the results, you might consider describing more general behavioral features such as success rate.

This has been done

12-     Results, section 3: 'even without preparation': please clarify what you mean by this

This has been done

13-     Results, section 4: 'Reward magnitude modulates BOTH SEF activity and pupil diameter

This has been done

14-     I would suggest to uniformly use pupil size or diameter but not both.

This has been done

15-     The authors will want to use the word CUE instead of hint.

This has been done

**Reviewer #3:** In this manuscript, the authors address the role that the supplementary eye fields (SEF) may play in relating to pupillary responses during a cognitively demanding task. The work is done in macaque monkeys, which is an important animal model to help establish the underlying neural mechanisms linking cognitively demanding tasks to pupil dilation, which is commonly observed in humans. Neural activity in the SEF is inferred by measures of cerebral blood volume (CBV) via functional ultrasound, a technique in which the authors are world leaders. Measuring SEF activity via CBV is akin to functional neuroimaging in some ways, with the exception that it comes with a better temporal resolution. The authors also manipulate reward levels to further examine the relationship between SEF-CBV and pupil dilation.

We sincerely thank the reviewer for his/her positive support to publish this study.

There are a number of positives to the manuscript. The experiments were conducted with great skill, and the authors present largely common trends across two animals. Further, the authors identify a gap in knowledge in the literature, and have found interesting distinctions between what was found in the SEF (which did relate to the pupil) versus the nearby anterior cingulate cortex (which did not). The paper is also largely well written, although I do have a number of comments below which I think may help convey the core message more clearly.

Main points

While I really enjoyed this manuscript, I struggled with whether it is fundamentally about establishing the relationship between SEF-CBV activity and pupillary responses (implied in the abstract) or about linking SEF activity to cognitive effort (implied by the title). My view is that the paper is more strongly positioned as the former.

The remark is well taken. We have proposed a new title that we hope better reflects the analysis and conclusion of this version of the article, more positioned as the relationship between the CBV in the SEF and the pupillary responses.

“**Short and long-time scales covariations between pupil diameter and Supplementary Eye Field activity**”

Further, the notion of how cognitive effort can be dissociated from other related phenomena, like attention or motivation, is complicated, and I am not convinced that the manipulations of reward used here do indeed dissociate effort from attention/motivation. If the authors agree with my sense that the paper is about establishing the SEF as an additional frontal area influencing the pupil, then aspects of the title and introduction could change (e.g., the reward manipulation becomes another way to test the link between SEF activity and the pupil). Alternatively, if the authors feel that the paper is about cognitive effort, then I think they need to do a better job rationalizing the additional value of pupil measurements.

We agree with the reviewer that the paper is more about establishing a link between the SEF and the pupil and not specifically on cognitive effort. We decide to remove the topic of cognitive effort in the new title.

The authors do a good job establishing short- and long-term correlations between SEF-CBV values and the pupil. For the short-term correlations however, and specifically regarding the fact that higher SEF-CBV values and greater pupil dilation accompany anti-saccades, I do worry that these results may reflect a confound of more saccades made on anti- vs pro-saccade trials, due to the inclusion of error trials. Is it possible to redo the analyses shown in Fig 3 using only correctly-performed anti-saccade trials?

The authors would like to apologize for the misunderstanding. The analysis in Fig 3 was already done using only the correctly-performed pro- and anti-saccade trials. This has been clarified in the manuscript page 7 L.301-302.

In the variant of the anti-saccade trial used here, the orientation of the peripheral target conveys the instruction to generate a pro- or anti-saccade. This variant differs from that used both by the Schlags in their seminal SEF work, as well as by the Wang studies in humans, where a feature of the initial fixation point conveyed task instruction. The latter variant introduces a greater separation in time of task preparation (which precedes target onset) from the saccadic response. I was curious as to the rationale for the authors to choose the variant of the anti-saccade task that they did. I think it would also help readers if the authors were more explicit about the difference in their task from that used by the work cited previously (the rationale and distinctions from previous work could go in the first paragraph of Results).

We thank the reviewer for this very pertinent remark. Indeed, Schlags and Wang used a version of the task where the fixation point conveyed the information about the nature of the subsequent saccade. However, Schall and colleagues have already published a similar variant of the antisaccade task (Sato and Schall 2003). We decided to train our animals in this variant in order to ambition in future experiments to parametrically manipulate the perceptual difficulty of target selection on each trial (by varying the ratio (height/width). We have at this stage too little data including this manipulation in a single monkey.

The manuscript has been modified p5, L177 to L181.

“The task differs from the usual pro- and anti-saccade task where the information about the nature of the trial is conveyed by the fixation point and not by the peripheral target. We decided to train our animals in this variant in order to ambition in future experiments to parametrically manipulate the perceptual difficulty of target selection on each trial (by varying the ratio (height/width)).”

Upon first reading, I struggled a bit with aspects of Figure 2. Regarding panels 2c/g and 2d/h, it would help if the authors could convey when the task actually started, and whether this coincided with changes in luminance/overall illumination. Am I correct in presuming that the task started at around the 300s point?

A green vertical bar has been added to the Fig 2c/g and 2 d/h indicating the start of the task. When measured, no significant changes in luminance of overall illumination has been observed, as the vast majority of the screen remain grey during all the task.

Also, regarding panels 2i/j/k, please specify in the results narrative that these are no longer single-session data, but instead summarize data across a larger sample of days. This information is currently only available deep in the Figure legend for Fig 2 (as an aside, showing data across multiple experimental session would seem to contradict the title to the Figure 2 legend, which emphasizes that the data is single-session data; this could also be corrected).

We are sorry for this confusion. The text has been modified (Page 23, Legend Fig. 2) and the title of the legend was replaced by “**Example of one session for each monkey of the vascular and pupillary responses and multiple sessions co-variation of pupillary and vascular responses** »

In the second paragraph of the discussion, the authors reference "two principal explanations" for their data. The first explanation follows in the next sentence, but I was not clear about the second explanation. Indeed the next few paragraphs would seem to be about qualifying the bottom-up explanation (which I presume is the first "principal explanation"), and the absence of increased CBV in the ACC. I could not find the second explanation; is it the top-down influence mentioned in the final paragraph? Regardless, please clarify these explanations in the Discussion.

The text has been modified (p.11-12, l.620 to l.665).

“In the ACC, unlike the SEF, there was not even a tendency of heightened CBV modulation under conditions of cognitive effort. This finding is compatible with an earlier report showing that ACC neurons in the monkey are not selectively active during the countermanding of saccades, an operation assumed to involve cognitive effort and inhibition of action^38^. However, it stands in sharp contrast to a large body of literature, based on functional MRI imaging in humans, indicating that activation in the ACC is strongly heightened under conditions of effort^39–42^. Here we report that during a sustained execution of a demanding oculomotor task over more than one hour, the neuro-vascular coupling (as assessed by fast ultra-sound imaging, fUS) in the supplementary eye field (SEF) of macaques, progressively drops with time, correlating with a simultaneous drop in pupil size and performance. When changing reward ratio, intrinsic brain mechanisms might compensate for the progressive drop in cognitive performance by instantiating alternations of high and low performance. These variations in behavioral performance might be phase-locked to variations in attention- and perception-related information as shown in macaque frontal eye field (FEF) neuronal populations^43^ »

Minor points

It is admittedly a stylistic point, but I felt there were aspects of abstract and introduction that were perhaps overstated. Notions like "underestimated yet pivotal" may be stretching thing a bit, and one could argue that the "pivotal" nature hasn't been established in this manuscript, since there was no true causal manipulation via stimulation or lesioning. Further, while I agree it is important to address the role of the SEF in pupil responses, I don't think many would be overly surprised that SEF activity relates to the pupil (given results from the FEF and ACC); others perhaps simply haven't measured it. Similar concerns about "pivot" relate to the usage of terms like "critically involved"; these strike me as terms that are just too strong to apply for the current dataset.

We agree with the reviewer. Our fervour during the analysis and writing of the summary led us to some semantic excesses. We have modified the title and abstract to better reflect our contribution and conclusion in view of the literature already existing. Futhermore, the term “pivot” has been removed from the abstract.

Please define CBV on first use in the abstract

This has been done

3rd paragraph of Introduction "…suggesting valid qualities for ongoing…". Not sure "qualities" is the right word here. Do the authors mean that pupil dynamics can provide a "proxy for" or "reflection of" cortical processing?

Please consider adding references to: 1) The first paragraph of intro where it states that the dmPFC mediates arousal. 2) Second paragraph of intro; consider references for the SEF projections to the oculomotor nucleus, and references for how the ACC/SEF/FEF may modulate the olivary pretectal nucleus.

Some references has been added to the introduction for the dmPFC and the ACC/SEF/FEF might modulate the olivary pretectal nucleus.

p.3 l.99	: Ebitz, R. B. & Moore, T. Selective Modulation of the Pupil Light Reflex by Microstimulation of Prefrontal Cortex. *J. Neurosci. Off. J. Soc. Neurosci.* **37**, 5008–5018 (2017).

p.3 l.99 : Lehmann, S. J. & Corneil, B. D. Transient Pupil Dilation after Subsaccadic Microstimulation of Primate Frontal Eye Fields. *J. Neurosci. Off. J. Soc. Neurosci.* **36**, 3765–3776 (2016).

p.3 l.101 : Trommershäuser, J., Glimcher, P. W. & Gegenfurtner, K. R. Visual processing, learning and feedback in the primate eye movement system. *Trends Neurosci.* **32**, 583–590 (2009).

p.3 l.101 : Schall, J. Visuomotor Functions in the Frontal Lobe. *Annu. Rev. Vis. Sci.* **1**, 150724172340006 (2015).

p.3 l.104 : Leichnetz, G. R. Connections between the frontal eye field and pretectum in the monkey: An anterograde/retrograde study using HRP GEL and TMB neurohistochemistry. *J. Comp. Neurol.* **207**, 394–402 (1982).

p.3 l.104 : Gonzalo-Ruiz, A., Leichnetz, G. R. & Hardy, S. G. Projections of the medial cerebellar nucleus to oculomotor-related midbrain areas in the rat: an anterograde and retrograde HRP study. *J. Comp. Neurol.* **296**, 427–436 (1990).

p.3 l.104 : Ebitz, R. B. & Platt, M. L. Neuronal activity in primate dorsal anterior cingulate cortex signals task conflict and predicts adjustments in pupil-linked arousal. *Neuron* **85**, 628–640 (2015).

**Reviewer #4:** In an excellent paper, Claron and colleagues report results of a pupillometry and fUS study of macaca mulatta cortex activity while monkeys perform in a rewarded saccade / anti-saccade task. The authors show fairly convincingly that SEF activity correlates more strongly than do ACC and control regions with pupil dilation (otherwise linked with cognitive effort), and that both SEF signal and pupil diameter covary reliably with reward magnitude, cross-session dynamics, and response to anti- vs pro-saccade trials and errors - all hallmarks of cognitive effort.

We sincerely thank the reviewer for his/her very positive support to publish this study.

On the basis of these correlations, the Authors argue that SEF activity tracks cognitive effort, yet additional tests might provide stronger evidence. Presumably, for example, if animals exert more effort on a given trial, they would be more accurate and faster, or (better yet), faster controlling for accuracy and vice versa. As such, it would be nice to show that when SEF activity were higher on a trial, the animals performed better.

This is a very interesting point. The SEF activity, taken trial-by-trial (and realigned on the target presentation) seems to be a little less intense at the end of the session than it is at the start of a session. However, at this time, we have too little evidence to link this diminution in the SEF trial-by-trial activity to the performance of the animal on a given trial. We hope that, in future experimentations, especially with the manipulation of trial difficulty, we will be able to quantify the link between SEF activity and performance within a single trial.

Also, while greater SEF activity for higher incentive trials and for anti- vs pro-saccades are both consistent with the hypothesis that the SEF tracks cognitive effort, a more convincing test is whether the two dimensions interact in predicting SEF activity. An interaction is more convincing because, conceptually, these two dimensions are also correlated with arousal, independently, but high arousal is not the same thing as high effort. Effort, in contrast, is the conversion of motivation into action to overcome a challenge. Thus, as a result of effort, the effects of task difficulty will vary as a function of motivational state, and vice versa.

We thank the reviewer for his/her comment. We performed further analysis and compared pupil and fus responses for Pro and Antisaccades as seen below. A trend seems to be observable between rewards but nothing is conclusive if we took the intensity of the signal by making the integral, as described in the methods.

**Figure R8: Difference in the ΔCBV (1^st^ row) for the pro-saccade (left) and anti-saccade (right) for different reward levels (red: low, blue : medium and green : high). 2^nd^ row: same for the pupil diameter in function of the reward level.**

Also, it is unclear whether effort follows the same low-frequency trends (downward, cross-session slopes) in pupil dilation and SEF activity. If pupil dilation and SEF activity are trending downwards across a session and they are also tracking effort, does that mean that monkeys are performing progressively worse across a session? If not, that seems to complicate the main inference.

We thank the reviewer for his/her comment. As seen below, we performed an analysis on the correct answers for anti-saccade over the time-span of one session (*circa* 1 hour) and we showed that monkeys perform worse across a session. Each individual session has been fitted with a linear fit, and we have a slope of -0.15% per trial of successfulness in anti-saccades, with p = 0.002 (t-test compared to null slope).

**Figure R9 : Percent of successful anti-saccade across sessions for both monkey S. and G. in mean +/- s.e.m. The 1st trials (1 to ~100) are usually less successful than the others, the monkey is usually recalling the task at these trials. We observe a steady decrease in the performance (-1.5% every 10 trials) significantly different from a null slope (p = 0.002)**

Minor comments

- In the results section, the Authors state that they "observed a slight disengagement of monkeys when the task is high-cognitive demanding … for a lower reward…" but they do not state their evidence of disengagement

Between rewards, we did expect a difference between the numbers of correct anti-saccade during a block of reward magnitude. As figured in Figure 4.e., we did observe a significant decrease of correct anti-saccades rates suggesting a slight disengagement during the low reward block (in red) compared with normal-rewarded or high-rewarded block (in blue and green, respectively). We sincerely hope that figure responds to the reviewer question.

- In Fig. 3, it is unclear why the shaded region between the anti- and pro-saccade conditions stops at 0,325 sec for the pupil diameter and at 4,0 sec for the CBV measurement?

We are sorry for the confusion. For the pupil diameter variation, we choose 0.320 s corresponding to :

- *circa* 270 ms for the saccade onset and execution (longest time being the antisaccade with ~ 270 ms onset time, as described in Fig. 1)
- 50 ms being the fixation of the ROI before receiving a reward in the case of a correct trial.

It allows us to calculate the differences during movement preparation for pro- versus anti-saccades without contamination of extra processes due to the reward event.

For the CBV measurement, we took 4 sec. as it corresponds to the time for estimated HRF response function to return to the baseline.  This has been clarified in the manuscript. (p.8, l.363-365 and l.371-373)

- It wasn't clear why the Authors used a somewhat arbitrary threshold (the ratio of accuracy on anti- vs pro-saccades < 0.6) to retain trials for analyses. I would like to know the sensitivity of the results to this arbitrary threshold (would conclusions change if they used values like 0.8 or 0.4, for example? It's also unclear why this threshold was used in the first place. Importantly, if the Authors only analyse sessions in which monkeys perform worse on anti-saccade trials by definition, then they are ensuring a positive correlation between errors and trial difficulty in a way that one would expect to covary with cognitive effort. That is, they are intentionally leaving out sessions where the presumably more difficult anti-saccade trials are performed equally well or better, and these sessions might provide and these sessions might be more revealing about if and how the SEF tracks cognitive effort. Finally, the Authors should stipulate the amount of data not retained for analyses according to their thresholding.

We, again, thank the reviwer for its precious insight. Please find the answer at reviewer #2, point 4.

We, again, thank the reviwer for its precious insight. Please find the answer at reviewer #2, point 4.

**Figure R2: All Z-map for monkey S and monkey G for all sessions obtained by applying the Generalized Linear Model using task and events as input matrix.**
