# Supplementary material for: Covariations between pupil diameter and supplementary eye field activity suggest a role in cognitive effort implementation
Source: PLoS Biol. 2022 May 26;20(5):e3001654. doi: 10.1371/journal.pbio.3001654 (PMC9135265; doi:10.1371/journal.pbio.3001654)
Supplement: Attachment — Submitted filename: Claron_et_al_SEF_Pupille_resp_Reviewers2.docx [file pbio.3001654.s004.docx]

Rev. 1:

All my previous concerns have been addressed in great detail by the authors, I am therefore happy to recommend the manuscript for publication.

We would like to really thank the reviewer #1 for their support to publish the manuscript.

Rev. 2:

I command the authors for their revision. I now think that the manuscript quality is highly improved and ready for publication. I also think that the change in title and abstract enhances the impact of this work. Congratulations to the authors !

We would like to deeply thank the reviewer #2 for their support to publish the manuscript.

Rev. 3:

I have read the authors' response, and well as their revised manuscript. I appreciate the level of attention to my comments, and the efforts exerted by the authors to revise their manuscript. From my perspective, things are largely resolved, and I only have a few minor points.

We thank reviewer #3 for their positive comments.

The abstract mentions the ACC without any context as to why this may be important. I'd suggest either deleting mention of the ACC in the abstract, or emphasizing how the SEF results contrast with negligible ACC signal changes. The ACC is also not defined in the abstract, nor upon first use in the introduction (line 55).

As the reviewer suggested, we changed this part of the abstract to emphasize the role of the SEF compared to the role of the ACC. Please find below changes made in the text : “Anterior Cingulate Cortex (ACC), as CBV variations in the ACC seems to be negligible compared to CBV variations in the SEF” (l.34-38)

Line 106 to 107, "...in order to ambition in future…" sounds a bit strange. Consider "… so that future.."

The suggested modification has been done (l. 108-109)

Lines 114 to 116 indicates that all trials were used in the analyses, regardless of whether they were correctly or incorrectly performed. This seems to contradict their responses to one of my concerns (Reviewer 3, 3rd main point in original review). I think all that is required is some qualifying statement in lines 114-116 (e.g., a statement to the effect that all data was used, unless noted otherwise). As an example lines 196-197 indeed do specify that only correct trials were used for a particular analysis.

We apologized for this inaccurate explanation. For all the analyses, we took all trials unless stated otherwise.

“All trials were used for futher analysis regardless of their nature (pro-saccades versus anti-saccades, right vs. left) or whether they were successful or not, unless stated otherwise.”

We hope that the l.161 to l.163 answer the reviewer’s concern, and we slightly modified the text in order to better reflect datas used in this analysis

Line 154, "i.e." instead of "id est"?

This modification has been done, as suggested (l.160)

I appreciate that the authors have rewritten much of the Discussion, and for the most part It reads well. The exception are the four paragraphs from lines 286-297, which read more like bullet points summarizing findings from four sets of studies. This portion of the Discussion could be improved with some edits to establish a cohesive narrative.

We totally agree with the reviewer on this portion of the discussion. As requested, the discussion has been modified in order to establish a cohesive narrative.

Rare studies have been capable of further examination. Some reports from sporadic patients with focal lesions of SEF , have shown great difficulty for these patients across a range of saccadic tasks^25–28^ , as well as, changing from an initial saccade plan to an alternative one^29,30^. These results were confirmed by functional imaging of healthy individuals performing a change-of-plan saccadic task, resulting in enhanced SEF activity when subjects successfully changed their saccadic plans compared to following a predictive plan^31^. Finally, some studies in human neuroimaging experiments have related these voluntary controls of saccade plan to a more general influence in the context of speed-accuracy tradeoff experiments^32,33^. l.389-l.397

Rev. 4:

In their revision, Claron and colleagues address some concerns and also provide additional analyses to address questions about whether the SEF and pupil dynamics track cognitive effort or not. On re-review, I agree with their decision to shift the emphasis (at least in the title) away from discussing cognitive effort. While there appears to be at least a nice long-term covariation between pupil dilation and SEF activity, and while changes in reward levels and demand level (anti- vs pro-saccade) seem to have similar impacts on pupil and SEF dynamics, evidence that these two features map to cognitive effort is relatively weak.

We would like to thank the reviewer #4 for their comments.

Specifically, for example, there are no interactions between reward and demand level, as you might expect if these systems track the cognitive effort involved. There is no close, trial-by-trial coupling between task performance and pupil or SEF dynamics. The Authors do find that slow timescale performance drops off in the same way that pupil dilation and SEF response do, over trials, but this effect could also be explained by a general fatigue effect, or accommodation to the task over time that is not specific to changes in effort. In none of their analyses do the Authors show that a trial-level increase in SEF (because of, say, a temporary increase in endogenously or exogenously driven motivation levels) predicts trial-level changes in pupil dilation - much less that such changes predict better trial-level performance…

A new concern has also arisen for me, in the Authors' response to a question about exclusion criteria, that also impacts on whether we can infer anything about effort from this dataset. Previously, the Authors had excluded data where ratio of accuracy on anti-vs pro-saccades was less than 0.6. When asked to consider all data, their response revealed a striking difference in the pattern of pupillary and SEF dynamics on anti- vs pro-saccades between the two monkeys in this study. Namely, while monkey S shows the expected pattern (pupil dilates more and performance is worse, on average, for anti-saccade trials, for most sessions), monkey G shows a very different pattern (pupil mostly dilates more, on average for pro-saccade trials, for most sessions, and average cross-session performance ratio is closer to 1.0 - indicating more similar performance between anti- and pro-saccade trials). In fact, excluding what look to be two outlier sessions, in terms of the ratio of dilation to

anti- vs pro-saccade trials, monkey G appears to have, on average, a higher dilation to pro-saccade trials. What do the Authors make of the fact that, more often than not, monkey G's pupil dilates more for pro-saccade trials? In any case, monkey G's dilation patterns suggest that their dilation doesn't faithfully track effort because pro-saccade trials aren't harder that anti-saccade trials. On a related note, I would like to know what the Authors make of the huge difference in the ratio of pupil dilation on anti- vs pro-saccade trials for monkey G on the two sessions. What was so different about these sessions that could explain why the dilation ratio was so dramatically different than it was for all other sessions for monkey G?

We, again, thank the reviewer for their question. Indeed, a slight error was present in the previous graphic for the outliers (**p-values remain the same as it was only a plotting error**). We deeply thank the reviewer for their insight, and we propose here the corrected figure. (red triangle: Monkey S., blue lozenge : Monkey G.)

**Figure R1 : Corrected ex- figure R4 for pupil.**

This divergence between monkeys raises serious questions about what can be inferred about effort, because it is unclear - for monkey G at least - whether greater dilation reflects greater effort. The complications with monkey G's data make me wonder how results would differ if the Authors analyzed monkey S's data only and asked whether trial-by-trial pupil dilation and SEF response to demand level or reward level predict better performance. If there is no strong coupling between trial-level data and performance, I recommend removing inferences about cognitive effort from the abstract.

The abstract has been modified accordingly to the reviewer’s suggestions:

Furthermore, we directly manipulated pupil diameter and CBV in the SEF using reward modulations. These results bring a novel understanding of the physiological links between pupil and SEF, but it also raises questions about the role of Anterior Cingulate Cortex (ACC), as CBV variations in the ACC seems to be negligible compared to CBV variations in the SEF.

l.34-l.38

It is okay to speculate about it in the discussion, but it seems unwarranted to make claims about effort in the abstract when it is unclear how well the current experimental design is reliably sensitive to differences in effort. In fact, it seems that the design neither reveals reliable patterns of dilation / SEF activity across monkeys nor reliable patterns about effort from trial-to-trial.

We modified the abstract to better account for the reviewer's concern (the trial-to-trial co-variation). Please find, in the answer above, the modifications done on the abstract.

Instead, I think the abstract, and conclusions should be focused on the much more compelling inference that long-term changes in pupil dilation map to long-term changes in SEF activity, and that two seem more strongly coupled than do pupil dilation and ACC on this timescale.

The title has been modified. We hope that the new title better reflects the reviewer’s expectation.

“Covariations between pupil diameter and Supplementary Eye Field activity suggest a role in cognitive effort implementation”

The conclusion also has been slightly modified to better reflect the reviewer’s expectation

“As SEF activity and pupil are strongly correlated over large time scales, our results also allow us to conclude that within the medial frontal cortex of primates, aside from the ACC, the SEF may also play a role in the implementation of the arousal signals observed within autonomous networks.” l.480-l.484
